# Fold formation at the compartment boundary of *Drosophila* wing requires Yki signaling to suppress JNK dependent apoptosis

**DOI:** 10.1038/srep38003

**Published:** 2016-11-29

**Authors:** Suning Liu, Jie Sun, Dan Wang, Gert O. Pflugfelder, Jie Shen

**Affiliations:** 1 Department of Entomology, China Agricultural University, 100193 Beijing, China; 2 Institute of Genetics, Johannes Gutenberg-University, 55128 Mainz, Germany

## Abstract

Compartment boundaries prevent cell populations of different lineage from intermingling. In many cases, compartment boundaries are associated with morphological folds. However, in the *Drosophila* wing imaginal disc, fold formation at the anterior/posterior (A/P) compartment boundary is suppressed, probably as a prerequisite for the formation of a flat wing surface. Fold suppression depends on *optomotor-blind* (*omb*). *Omb* mutant animals develop a deep apical fold at the A/P boundary of the larval wing disc and an A/P cleft in the adult wing. A/P fold formation is controlled by different signaling pathways. Jun N-terminal kinase (JNK) and Yorkie (Yki) signaling are activated in cells along the fold and are necessary for the A/P fold to develop. While JNK promotes cell shape changes and cell death, Yki target genes are required to antagonize apoptosis, explaining why both pathways need to be active for the formation of a stable fold.

Epithelial folds contribute to morphogenetic movements and the separation of different cell groups, thus shaping the animal body^1^. For example, invagination of the *Drosophila* mesoderm is initiated by ventral fold formation^2^,^3^. Segmental and parasegmental grooves transiently appear in the trunk region of the *Drosophila* embryonic epidermis separating fields of cells of different fate^4^,^5^. A common but not the only mechanism of epithelial fold formation involves apical cell constriction and the acquisition of a bottle-like cell morphology^6^. By this mechanism tubes can be formed from epithelial sheets as in the development of tracheal and salivary gland primordia^7^. Folds also arise in postembryonic epithelia. In the *Drosophila* eye disc, differentiation depends on the progression of the morphogenetic furrow, a Hedgehog-dependent apical indentation of the eye field^8^,^9^. Apical and basal folds can also form at the borders which separate cell groups of different fate in the other imaginal discs of *Drosophila* larvae^10^,^11^,^12^. Folds can arise in the epithelia of all metazoa. In sea urchins, bottle cells have been shown to be required for invagination of the ectoderm^13^. In vertebrates, classical examples include the formation of the neural tube in chick^14^ and of the blastopore lip in amphibians^15^,^16^. Neuroectodermal grooves are also found during brain development in mouse and zebrafish^17^,^18^.

As outlined above, folds occur in many aspects of *Drosophila* development, making this species an excellent model organism to study mechanisms of fold formation. *Drosophila* has provided information on the molecular underpinnings of the required cell shape changes in various developmental paradigms. The best studied system is gastrulation which was investigated at the levels of genetics, cell biology and biophysics and which, therefore, can serve as a benchmark for studies in other systems^19^,^20^,^21^,^22^. In *Drosophila* gastrulation, the secreted protein Folded gastrulation (Fog) is central to inducing apical constriction of the invaginating cells^23^,^24^ but is dispensable in several other epithelial folding processes. In the *Drosophila* embryonic ectoderm, the formation of segmental grooves was shown to be controlled by *engrailed* (*en*) expression in boundary cells as well as by Hedgehog (Hh) and Wingless (Wg) signaling^4^. These segment polarity genes are not involved in specifying the position of nearby parasegmental grooves; Wg signaling is required, however, in a non-instructive, permissive role^5^. Formation and progression of the morphogenetic furrow in the larval eye disc is controlled by Hh and Decapentaplegic (Dpp). Induction of the cell adhesion molecule Cad86C by Hh and Dpp may be one of the mechanisms that effects cell shape changes in this tissue^25^. The way folds form, thus, can differ with regard to molecular and biomechanical requirements even within one epithelium. For example, in *Drosophila* gastrulation, the ventral furrow forms by apical constriction whereas the dorsal folds arise by a basal shift of the adherens junctions^26^.

In the larval wing disc pouch (the future wing blade), the normal, graded expression of Dpp and Wg does not instruct folding but rather is involved in maintaining the appropriate position-specific cell shape^27^. In the columnar main epithelium, loss of Dpp signaling causes extrusion of cells correlated with loss of the apical microtubule web^28^,^29^. Similarly, loss of Dpp targets Optomotor-blind (Omb) or Spalt lead to retraction of cells toward basal membrane^30^,^31^. Dpp signaling cell-autonomously promotes and maintains the elongated columnar shape of wing disc cells by regulating Rho1 and the regulatory light chain of non-muscle myosin II^32^. Wg signaling cell-autonomously promotes and maintains the columnar shape of wing disc cells through maintaining Vestigial (Vg) expression^33^. The Wg gradient, centered on the D/V boundary, instructs similarly shaped gradients of DE-cadherin concentration and apical cell circumference (high and constricted, respectively, close to the D/V boundary)^34^. The loss of Adenomatous polyposis coli (APC), a negative regulator of Wg signaling, leads to apical constriction and invagination independent of its effect on the DE-cadherin level. Wg, too, acts by activation of Rho1 and Myosin II^35^.

The folds which separate parasegments in the *Drosophila* embryonic ectoderm separate fields of cells that are related by lineage (compartments)^36^. But even in the absence of lineage restriction, groups of epithelial cells differing in gene expression and fated to develop into different structures tend to be separated by a fold. For instance, in the wing imaginal disc, which gives rise to adult notum, hinge, and wing blade, several folds orthogonal to the proximo-distal axis separate gene expression domains without being lineage restriction boundaries^37^,^38^. The most distal of these, the blade/hinge fold develops under control of the Omb-related T-box transcription factors Dorsocross (Doc)^39^. The proximal notum/hinge fold requires the complementary expression of Omb in hinge and Iroquois complex (Iro-C) in notum^40^. In contrast, the A/P compartment boundary is not associated with a fold and remains morphologically inconspicuous throughout development^41^, even though it derives from the corresponding infolded parasegmental boundary in the embryonic ectoderm^42^. It is conceivable that fold formation was selected against because of the structural requirement for the adult wing as a flight appendage. Indeed, fold formation is actively suppressed by a genetic program. Omb which is expressed in most of the pouch^43^ is required to maintain the normal epithelial structure at the A/P boundary. Reduction of Omb level in the pouch causes an apical morphogenetic defect at the A/P boundary due to contraction of cells along their apical-basal axis^44^. We here investigate the mechanisms of boundary fold formation elicited by Omb loss. We found that A/P fold formation is dependent on activation of JNK signaling induced by loss of *omb*. Loss of *omb* also induced Yki activity which promoted cell survival and attenuated the pro-apoptotic activity of JNK. Our results reveal a network of signaling pathways induced by loss of *omb* that controls cell shape and ensures cell survival of folded cells at the A/P boundary.

## Results and Discussion

We and others have shown before that Omb prevents aberrant apical fold formation at the A/P boundary^44^,^45^. When Omb is directly or indirectly repressed in the P compartment, the A/P boundary of the wing develops a deep apical fold in the larval wing disc and a cleft in the adult wing^44^. We here use this model system to investigate the mechanisms of boundary fold formation under three genetic manupulations, ptc-Gal4 UAS-tkv, en-Gal4 UAS-omb-RNAi, and nub-Gal4 UAS-omb-RNAi (Fig. S1).

### JNK is ectopically activated to initiate A/P fold formation

**Figure 1:**
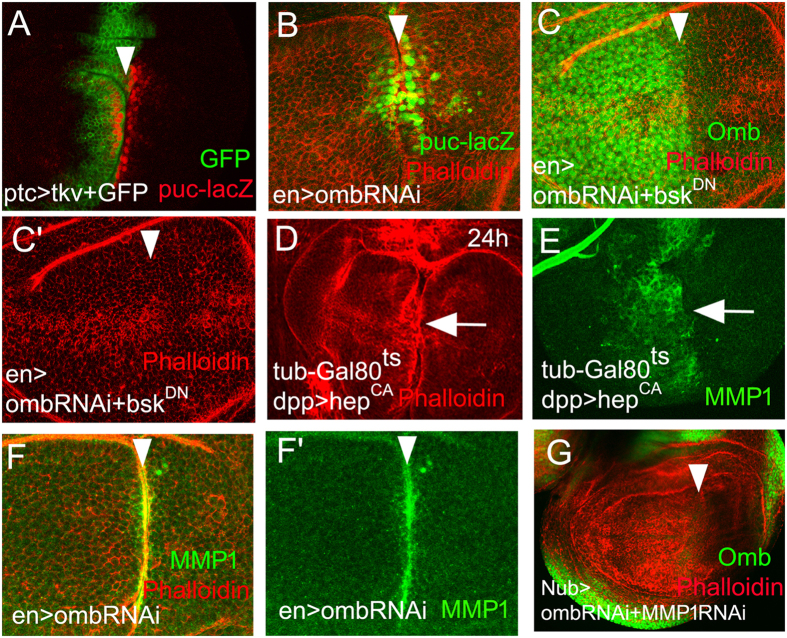
JNK signaling is necessary for A/P fold formation. (**A**,**B**) puc-lacZ was ectopically activated at the A/P fold. (**C**) Repression of JNK signaling by co-expressing a dominant negative form of JNK (bsk^DN^) suppressed the A/P fold formation. Anti-Omb staining (green) demonstrates the efficiency of the posterior knock-down. Activation of JNK signaling by hep^CA^ for a short duration induced extended folding (**D**) and Mmp1 expression (**E**) in the dpp-Gal4 domain. (**F**,**F’**) Focused induction of Mmp1 expression symmetrically on both sides of the A/P fold in en>omb-RNAi wing disc. (**G**) Co-expressing Mmp1-RNAi suppressed A/P fold formation. Arrowheads point at the position of the A/P boundary.

To investigate potential effectors downstream of Omb, we monitored the expression or activity of candidate targets. JNK signaling is an important pathway in the regulation of wing disc morphogenesis^46^,^47^. We monitored JNK pathway activity by monitoring transcription of the JNK target gene *puckered* (*puc)*^48^. In both ptc>tkv and en>omb-RNAi wing discs, *puc* was activated along the A/P fold (Fig. 1A,B). *puc* was initially activated in cells adjacent to the fold in early-mid L3, then its expression extended further into the A and P compartments (Fig. S2). These data indicate that JNK signaling is activated in the process of A/P fold formation.

We next asked whether JNK activation is required for A/P fold generation. Co-expressing omb-RNAi and a dominant negative form of JNK, *bsk*^*DN*^ 49, was sufficient to suppress A/P fold formation (Fig. 1C).

This suggests that activation of JNK signaling is required in this process. To test for sufficiency of JNK activation for A/P fold formation, we activated JNK by expressing *hep*^*CA*^ (encoding a constitutively active form of JNKK^50^) for a short duration controlled by dpp-Gal4 and tub-Gal80^ts^ (continuously activation of hep^CA^ induced severe apoptosis thereby disturbing observation of cell morphology). Under these conditions folds occurred throughout the dpp-Gal4 expression domain (Fig. 1D). The broad anterior activation of JNK signaling did not lead, however, to a discrete A/P fold.

The matrix metalloproteinase 1 (Mmp1) is induced by ectopic activation of JNK during morphological reorganization of epithelia^51^,^52^,^53^,^54^,^55^. When the JNK pathway was activated for 24 h in the dpp-Gal4 expression domain, Mmp1 was broadly induced anterior to the A/P boundary, similar to the plexus of epithelial folds observed under these conditions (Fig. 1E). However, in en>omb-RNAi wing discs, Mmp1 accumulated in a discrete stripe of A/P fold cells on both sides of the fold (Fig. 1F and F’). Co-expression of omb-RNAi and Mmp1-RNAi with nub-Gal4 rescued the A/P fold with full penetrance (Fig. 1G). Uniform reduction of *omb* expression on both sides of the A/P boundary, like posterior *omb* reduction, leads to A/P fold formation (Fig. S1E and F).

However, expression of *Mmp1* with dpp-Gal4 along the A/P boundary did not generate a fold (Fig. S3). These data suggests that either additional gene expression changes, induced by the loss of *omb*, are necessary for A/P fold formation or that *Mmp1* must be induced on both sides of the A/P boundary.

### Yki-Diap1 signaling is activated parallel to the JNK pathway

**Figure 2:**
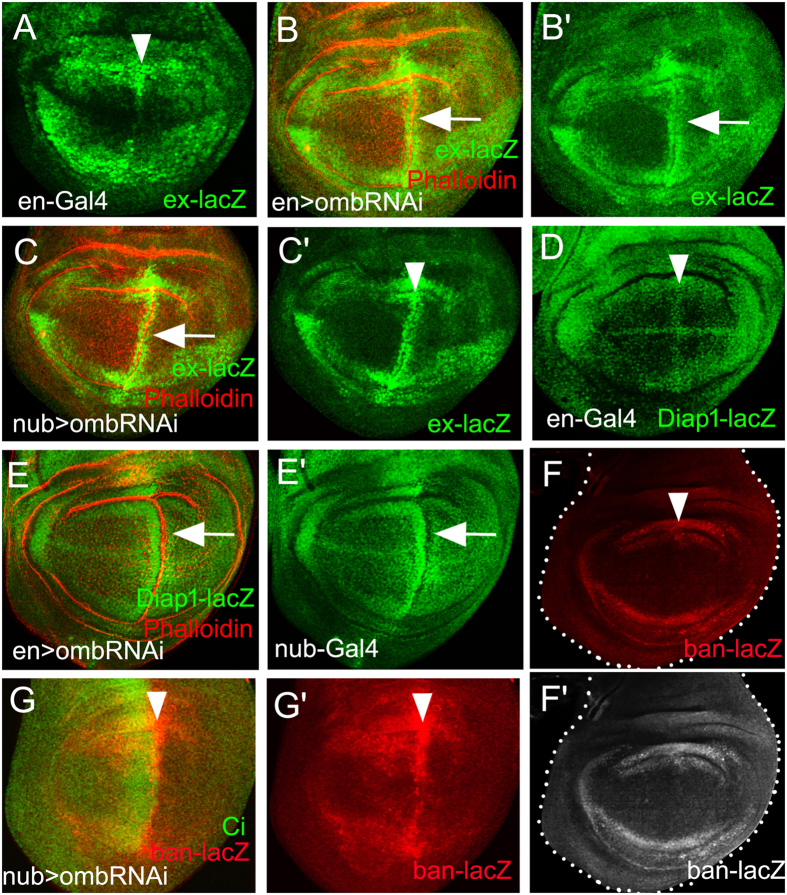
Activation of Yki target genes in *omb* hypomorphic wing discs. (**A**) Control experiment to show the normal ex-lacZ expression. (**B,C**) ex-lacZ was upregulated along the A/P fold in discs in which *omb* was knocked down in the posterior compartment (**B**) or in the entire pouch (**C**). (**D**) Control experiment to show the normal Diap1-lacZ expression. (**E**) Diap1-lacZ was upregulated along the A/P fold. (**F**) Control experiment to show the normal ban-lacZ expression. (**G**) ban-lacZ was ectopically activated at the A/P fold.

It has been reported that Dpp and Wg repress JNK signaling to maintain survival of wing pouch cells. Loss of either Dpp or Wg signaling activates JNK-dependent apoptosis in the wing pouch^50^. However, *omb* knock-down did not induce apparent apoptosis at the A/P boundary^44^, although JNK signaling was activated (Fig. 1A and B). We assume that the apoptosis pathway is repressed in this case. Yki signaling can be induced by the JNK pathway^56^. Yki targets such as *Death-associated inhibitor of apoptosis 1* (*Diap1*) and the microRNA *bantam* (*ban*) can repress apoptosis^57^,^58^,^59^. We analyzed transcription of the Yki target *expanded* (*ex*^60^ and observed that ex-lacZ was up-regulated at the A/P fold generated by en>omb-RNAi (Fig. 2B), suggesting an activation of Yki signaling during A/P fold formation. This was confirmed in nub>omb-RNAi wing discs (Fig. 2C). Upregulation at the A/P fold was also observed for *Diap1* (Fig. 2E). The Yki target *ban* is suppressed by Omb in the medial wing discs^61^. Consistently, *ban* was up-regulated in the medial wing disc of nub>omb-RNAi larvae, with the strongest enhancement along the A/P boundary (Fig. 2G). Therefore, during A/P fold generation, *ban* and *Diap1* were both activated and could suppress potential apoptosis induced by changes in cell shape and JNK activation.

In order to determine whether the Yki targets were induced as a consequence of JNK signaling, we co-expressed bsk^DN^ and omb-RNAi in the nub-Gal4 domain. When JNK pathway and A/P fold were suppressed, ex-lacZ expression was still activated at the A/P boundary (Fig. S4A). This also held for *Diap1* and *ban* expression (Fig. S4B and C). This suggests that in A/P fold formation Yki can be activated even when the JNK pathway is blocked. Yki activation, thus, appears to occur parallel to JNK signaling and is not sufficient for fold formation.

**Figure 3:**
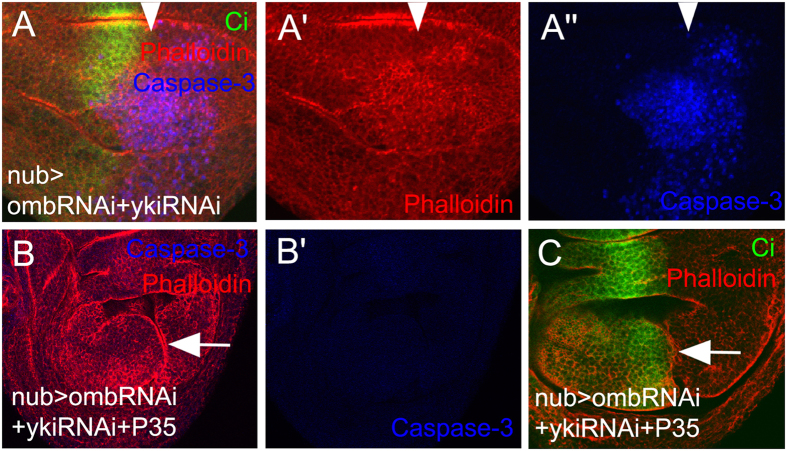
Suppression of apoptosis is required for fold formation. (**A-A”**) Co-expressing omb-RNAi and yki-RNAi by nub-Gal4 suppressed the A/P fold formation but induced severe apoptosis. (**B**,**B’**) Whenapoptosis was suppressed by co-expressing p35 an A/P fold was generated. (**C**) The fold generated in nub>[omb-RNAi + yki-RNAi + p35] wing discs extended along the A/P boundary (anterior compartment marked by Cubitus interruptus (Ci) expression, green).

To test whether the suppression of cell death by Yki signaling is required for *omb*-loss induced A/P fold formation, yki-RNAi was co-expressed with omb-RNAi in the nub-Gal4 domain. As shown in Fig. 3A-A”, co-expressing yki-RNAi was sufficient to suppress the formation of A/P fold. Severe cell death occurred in this double knock-down. When p35 was co-expressed with omb-RNAi and yki-RNAi in the nub-Gal4 domain to inhibit apoptosis, cell death was effectively suppressed (Fig. 3B and B’) and the A/P fold appeared again (Fig. 3B and C). These data indicate that Yki signaling is required for A/P fold by ensuring cell survival.

Generally, abnormal activation of the JNK pathway induces apoptosis. For instance, expression of activated tumor genes or mutation in tumor suppressor genes lead to JNK-induced cell invasion and apoptosis^55^,^62^,^63^,^64^. However, in *omb*-knocked-down wing discs, the activation of JNK pathway did not cause cell death along the A/P fold^44^. We suggest that cell death is suppressed by the simultaneous induction of a cell survival pathway. Yki has an important role in promoting cell survival by driving the expression of downstream genes such as *Diap1* and *ban*^65^. We found these genes upregulated along the A/P fold (Fig. 2). This suggests that Yki antagonizes apoptosis along the fold. Previous studies identified JNK as a promoter of Yki activity in the wing disc^56^,^63^,^66^. But this regulatory relationship is not absolute^62^,^63^,^67^. We found that co-expression of omb-RNAi and bsk^DN^ had no effect on *ex, Diap1,* and *ban* expression (Fig. S4). This indicates that, at the A/B boundary, Yki is activated parallel to JNK signaling.

## Methods

### *Drosophila* stocks

The transgenes used were as follows: en-Gal4, nub-Gal4^68^, dpp-Gal4, ptc-Gal4, UAS-tkv, UAS-CD8-GFP, UAS-GFP, UAS-omb-RNAi^44^, tubP-Gal80^ts^^69^, UAS-MMP1^70^, UAS-MMP1-RNAi^55^, UAS-p35, UAS-hep^CA^, UAS-bsk^DN^, UAS-yki-RNAi (TsingHua Fly Center). Enhancer trap lines were hh-lacZ^71^, puc-lacZ, ex-lacZ, diap1-lacZ^60^, and ban-lacZ^72^. Stocks, if not mentioned otherwise, were obtained from the Bloomington stock center.

Larvae were raised at 25 °C. For efficient expression of RNAi transgenes, larvae were raised at 29 °C. Larvae containing Gal80^ts^-Gal4 combinations were raised at 18 °C and then were shifted to 29 °C that allows GAL4 to function and activate transcription of UAS controlled transgenes.

### Immunohistochemistry

Dissected wing imaginal discs were fixed and stained with antibodies according to the standard procedures. The primary antibodies used were: rabbit anti-Omb, 1:1000; mouse and rabbit anti-beta-galactosidase, 1:2000 (Promega); rabbit anti-caspase3, 1:200 (Santa Cruz), rat anti-2A1 (Ci), 1:200 (Developmental Studies Hybridoma Bank, DSHB); and mouse anti-Mmp1, 1:200 (DSHB). Secondary antibodies used were goat anti-rabbit DyLight 488, goat anti-rat DyLight 488, goat anti-rabbit DyLight 549, goat anti-mouse DyLight 488, goat anti-rabbit Cy3, and goat anti-rabbit Cy5, were diluted 1:200 (Agrisera). Actin was visualized with Rhodamine- phalloidin, 1:1000 (Cytoskeleton). Images were collected using a Leica TCS SP2 AOBS confocal microscope.

## Additional Information

**How to cite this article**: Liu, S. *et al*. Fold formation at the compartment boundary of *Drosophila* wing requires Yki signaling to suppress JNK dependent apoptosis. *Sci. Rep.* **6**, 38003; doi: 10.1038/srep38003 (2016).

**Publisher's note:** Springer Nature remains neutral with regard to jurisdictional claims in published maps and institutional affiliations.

## Supplementary Material

Supplementary Information
