# Supplementary material for: Fold formation at the compartment boundary of Drosophila wing requires Yki signaling to suppress JNK dependent apoptosis
Source: Sci Rep. 2016 Nov 29;6:38003. doi: 10.1038/srep38003 (PMC5126554; doi:10.1038/srep38003)
Supplement: Supplementary Information [file srep38003-s1.pdf]

Fold formation at the compartment boundary of *Drosophila* wing requires Yki signaling to suppress JNK dependent apoptosis

Suning Liu[1], Jie Sun[1], Dan Wang[1], Gert O. Pflugfelder[2], and Jie Shen[1]*

1. Department of Entomology, China Agricultural University, 100193 Beijing, China

2. Institute of Genetics, Johannes Gutenberg-University, 55128 Mainz, Germany

*Corresponding author. E-mail: shenjie@cau.edu.cn, Tel (0086) 10 627 32384

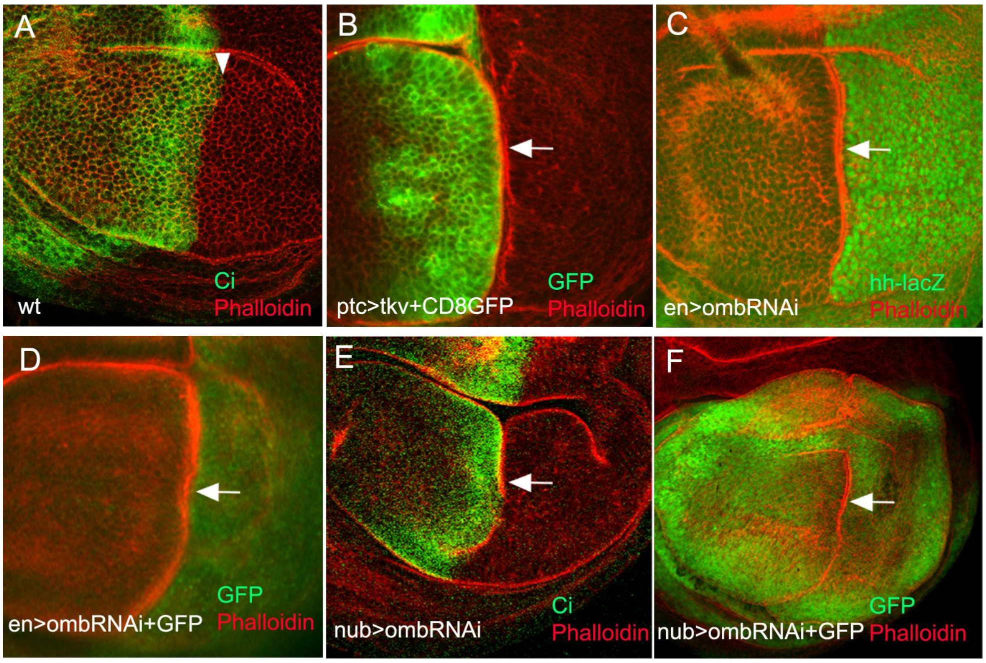

Figure S1. Suppression of Omb induces A/P fold formation at the A/P boundary.

(A) Even surface of the wing disc pouch epithelium across the A/P boundary (arrowhead) in wild type. (B) Indirect suppression of posterior *omb* by anterior expression of the Dpp receptor Thickveins (Tkv) induces a fold formation (arrow) at the A/P boundary. (C and D) Expressing omb-RNAi in the posterior compartment induces an A/P fold (arrow). (E and F) Expressing omb-RNAi in the entire pouch region induces an A/P fold (arrow).

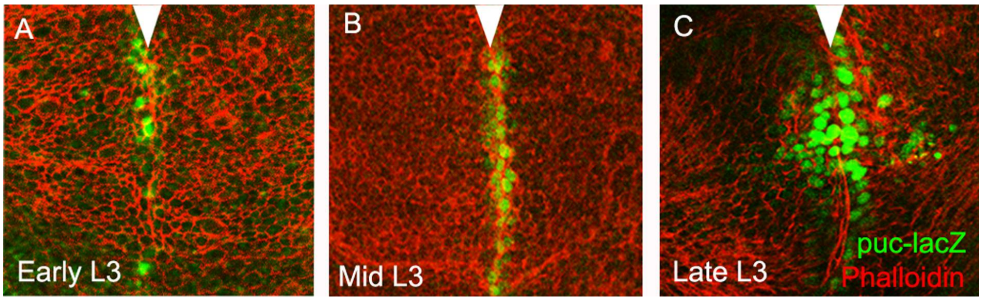

Figure S2. Expansion of puc-lacZ expression at the A/P fold during the third larval instar.

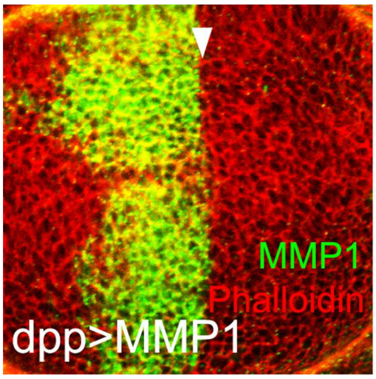

Figure S3. MMP1 is not sufficient for A/P fold induction.

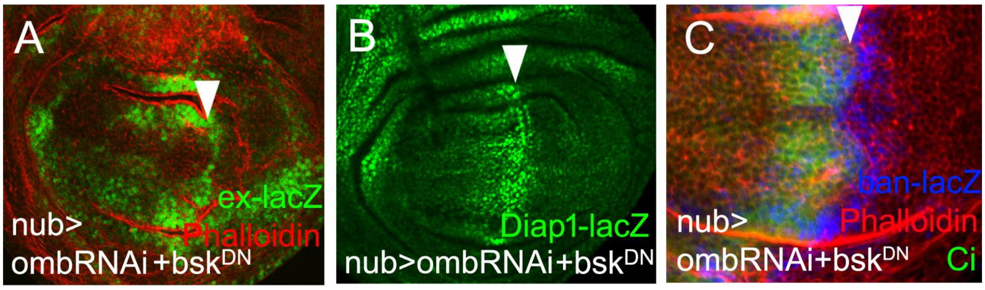

Figure S4. Yki-Diap1 signaling is activated parallel to JNK signaling.

(A) Co-expressing omb-RNAi and bsk^{DN} by nub-Gal4 suppressed A/P fold formation, but not ex-lacZ expression at the A/P boundary. (B, C) Co-expressing omb-RNAi and bsk^{DN} did not suppress Diap1-lacZ (B) and ban-lacZ (C) expression at the A/P boundary.

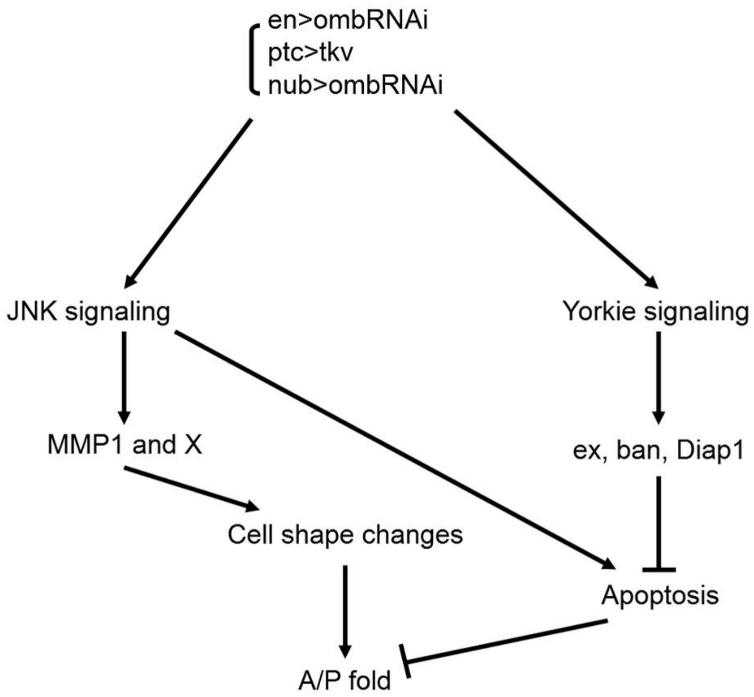

Figure S5. Model exploring the connection of signaling pathways in omb loss-induced A/P fold formation

Posterior or uniform reduction of *omb* expression in the wing disc pouch induces JNK signaling and Yki activity in cells flanking the A/P boundary. JNK signaling is sufficient to induce apoptosis, which is, however, repressed by the induction of Yki target genes such as *Diap1* and *ban* at the A/P boundary. Suppression of apoptosis allows development of the A/P fold.
